# *dFatp* regulates nutrient distribution and long-term physiology in *Drosophila*

**DOI:** 10.1111/j.1474-9726.2012.00864.x

**Published:** 2012-12

**Authors:** Alyson Sujkowski, Samantha Saunders, Martin Tinkerhess, Nicole Piazza, Joanna Jennens, Lindsey Healy, Li Zheng, Robert Wessells

**Affiliations:** Department of Internal Medicine, Institute of Gerontology, University of Michigan Medical SchoolAnn Arbor, MI, USA

**Keywords:** aging, cardiac declines with age, drosophila, life span, senescence, training

## Abstract

Nutrient allocation and usage plays an important part in regulating the onset and progression of age-related functional declines. Here, we describe a heterozygous mutation in *Drosophila* (*dFatp*) that alters nutrient distribution and multiple aspects of physiology. *dFatp* mutants have increased lifespan and stress resistance, altered feeding behavior and fat storage, and increased mobility. Concurrently, mutants experience impairment of cardiac function. We show that endurance exercise reverses increased lipid storage in the myocardium and the deleterious cardiac function conferred by *dFatp* mutation. These findings establish a novel conserved genetic target for regulating lifespan and physiology in aging animals. These findings also highlight the importance of varying exercise conditions in assessing aging functions of model organisms.

## Introduction

Fatty acids (FA) are a significant dietary component in diverse animal species, necessary to form precursors for cell membranes and chemical messengers (Grammatikos *et al*., 1994). In addition, FA act as signaling molecules to regulate multiple physiological processes, including electrical impulse transmission, signal transduction, and gene expression (Pegorier *et al*., 2004).

Dysregulation of fatty acid levels or activities contributes to several important pathologies that are associated with the aging process, particularly the interrelated network of obesity, cardiovascular disease, and type 2 diabetes that comprises the metabolic syndrome (Ford, 2010). Extensive epidemiological evidence exists that high-fat diets and obesity can induce pathological processes leading to increasing atherosclerosis, inflammation, hypertension, and dyslipidemia (Short *et al*., 2009; King *et al*., 2010). Additionally, lipids are observed to accumulate during aging in cardiac myocytes (Slawik & Vidal-Puig, 2006). These changes promote insulin resistance and cardiovascular disease, which, in turn, are enhanced by changes brought about through normal aging.

Aging per se causes reduction in cardiac performance, both indirectly through alterations in vascular health and directly through changes in myocardial physiology (Lakatta, 2003). These changes are mediated in part by alterations in fatty acid usage. During human aging, a decrease in fatty acid usage in the myocardium has been observed (Kates *et al*., 2003), suggesting a decrease in FA-derived ATP production, perhaps leading to energetically compromised hearts. These changes are mediated in part by age-related changes in myocardial gene expression of factors essential for beta-oxidation of FA (Weindruch, 2003). In turn, changes in cardiac gene expression and physiology are modulated by dietary intake, because they can be reversed by caloric restriction (Dhahbi *et al*., 2006).

Dysregulation of fatty acid uptake and usage is therefore a potentially important factor in the etiology of several health problems prevalent in the elderly population. One potential area of regulation that could be targeted for potential interventions is fatty acid transport. Uptake of long-chain FA (LCFAs) is thought to occur by two different mechanisms: diffusion and protein-mediated transport (Ehehalt *et al*., 2006). Several proteins have been identified that have a high affinity for LCFAs. These include FABPpm, FAT/CD36, and the fatty acid transport protein (FATP) family (Stahl, 2004).

Several mammalian FATPs have been identified, and recent work suggests that these proteins may not influence cellular uptake of FA by direct transport, but instead may regulate the usage by virtue of their intrinsic acyl-CoA synthetase activity (Ehehalt *et al*., 2006). In addition to regulating the concentration of FA available for β-oxidation, such activity also facilitates passive transport mechanisms by altering the gradient of fatty acid availability inside the cell, a process known as vectorial acylation (Black & DiRusso, 2007). The six mammalian FATP family members, despite a high degree of sequence similarity, exhibit different functional properties by virtue of their different expression patterns (Stahl, 2004). FATP6 is the predominant FATP in the mammalian heart, while FATP1 is expressed in the heart, skeletal muscle, as well as highly in adipose tissue (Hirsch *et al*., 1998; Stahl *et al*., 2001; Stahl, 2004).

In cultured fibroblasts, overexpression of mouse *FATP1* leads to an increase in long-chain fatty acid uptake (Schwenk *et al*., 2010). Conversely, disrupting the *FATP1* homologue *fat1* in yeast *Saccharomyces cerevisiae* impairs long-chain fatty acid uptake (Zou *et al*., 2002). FATP1 has been shown to possess acyl-CoA synthetase activity, and the ability of FATP1 to regulate fatty acid uptake is dependent on such activity (Richards *et al*., 2006).

Flies have previously been shown to be effective models for the study of aging (Hughes & Reynolds, 2005), fat metabolism (Teleman, 2010), adult cardiac function (Piazza & Wessells, 2011), and endurance exercise (Piazza *et al*., 2009). Endurance exercise has long been known to improve cardiovascular function in vertebrates (Kemi & Wisloff, 2010), and flies also respond to endurance training by improving cardiac output and stress resistance at advanced ages (Piazza *et al*., 2009). This, combined with the unparalleled genetic tools available for studies in the fly, presents an important opportunity to examine the impact of FATP family genes on metabolism and function at varying ages and varying degrees of exercise.

The genome project has identified three FATPs in flies, based on sequence homology. The *Drosophila dFatp* gene encodes a product closely related to *hsFATP1* (Hirsch *et al*., 1998). Here, we examine the role of this FATP ortholog in metabolism, lifespan, stress resistance, mobility, and cardiac performance. We find that flies carrying one copy of a loss-of-function mutation in the *dFatp* gene exhibit increases in triglyceride storage and circulating free FA, with a concurrent reduction in feeding behavior. These metabolic and behavioral alterations lead to an extended lifespan as well as improved resistance to multiple stresses. However, *dFatp* mutants exhibit defects in cardiac performance, which can be selectively reversed by an endurance exercise program.

## Results

### Gene locus

We obtained a line containing a gene trap insertion in the *dFatp* locus *dFatp*^*K10307*^ (Fig. S1A). This insertion is homozygous lethal, but heterozygotes are viable with a 72% reduction in mRNA expression (Fig. S1B). We attribute the phenotypes observed in *dFatp*^*K10307*^ heterozygotes to the *dFatp* locus for the following reasons: (i) no significant alteration in mRNA expression is seen in any of three neighboring genes (Fig. S1C–E); (ii) the insertion is lethal over deletions in the region, but not over single-gene null mutants in the region other than *dFatp* itself; and (iii) precise excision of the insertion restores phenotypes to wild-type. These flies will be referred to as *dFatp* revertants. An inducible RNAi construct targeting the *dFatp* gene product provides an effective knockdown with a ubiquitous *da-gal4* expression driver. Ubiquitous induction of this construct throughout development generates a semi-lethal phenotype with an 83% reduction in *dFatp* mRNA levels (Fig. S1B).

To avoid complicating effects from mutations contained on balancer chromosomes, we have performed all experiments described below on flies in which the *dFatp*^*K10307*^ insertion has been backcrossed to the starter line in which the original insertion was isolated. The insertion was backcrossed 10 times, using the *w*^*+*^ associated with the insertion as a selectable marker. *y*^*1*^*w*^*67c23*^;*dFatp*^*K10307*^*/+* flies will henceforth be referred to as *yw;dFatp*^*het*^ and compared to background and age-matched *y*^*1*^*w*^*67c23*^ flies. *y*^*1*^*w*^*67c23*^ flies will be referred to as *yw*. To account for the possibility that the effects of the *dFatp*^*k10307*^ insertion are specific to the *y*^*1*^*w*^*67c23*^ background, we also outcrossed the *dFatp*^*k10307*^ insertion into the *y*^*1*^*w*^*1*^ background using similar methods, except only one round of additional backcrossing was employed in this case. We separately performed a single outcross of the *dFatp*^*k10307*^ insertion into the *white Canton S* (*w[cs]*) background. *w[cs]* is the common *Canton S* background with a white mutation backcrossed in (Cook-Wiens & Grotewiel, 2002). This stock allows for transgenes marked with a w^+^ to be backcrossed into a healthy background for physiological experiments. The aforementioned outcrosses will henceforth be referred to as *y*^*1*^*w*^*1*^;*dFatp*^*het*^ and *w[cs]*;*dFatp*^*het*^. *y*^*1*^*w*^*1*^*;dFatp*^*het*^ is compared to *y*^*1*^*w*^*1*^, while *w[cs];dFatp*^*het*^ is compared to F1 progeny of a cross between *dFatp* revertant and *w[cs]*. Progeny of this cross are controls for the hybrid background created by the single outcross and are referred to as *w[cs];dFatp* revertant.

*dFatp* expression has been reported to be enriched in the fat body, heart, and in multiple regions in the gut (http://www.flyatlas.org/). Here, we take advantage of the reduction in *dFatp* expression conferred by the *dFatp*^*K10307*^ insertion to examine the resulting metabolic and physiological effects.

### Triglyceride storage and feeding rate

**Fig. 1:**
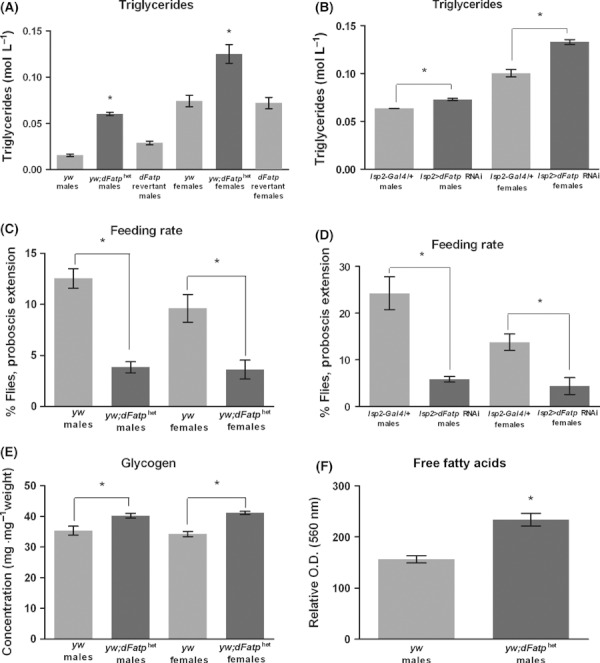
Reduction in *dFatp* expression increases energy storage while lowering feeding rate. (A) Triglyceride levels of *yw;dFatp*^*he*t^ flies are compared to *yw* background controls. Mutants have significantly higher levels of TAG (*t*-test: *P* < 0.001, males, *P* = 0.013, females). *dFatp* revertant flies return to wild-type triglyceride levels (*t*-test *P* = 0.646). (B) Knockdown of *dFatp* in fat (*lsp2* *>* *dFatp* RNAi) causes an increase in TAG compared to control flies (*t*-test: *P* = 0.013 males and 0.002 females). (C) *yw;dFatp*^*he*t^ mutants have a significantly reduced feeding rate compared to *yw* background controls (*t*-test: *P* = 0.022 males and *P* = 0.016 females). (D) Knockdown of *dFatp* in fat (*lsp2* *>* *dFatp* RNAi) causes a significant decrease in feeding rate (*t*-test: *P* = 0.006 males and *P* = 0.02 females). (E) Glycogen levels in total fly homogenate are significantly higher in both male and female mutants (*t*-test: *P* = 0.027 males and *P* < 0.001 females). (F) Free fatty acid levels in circulating hemolymph are significantly higher in male *yw;dFatp*^*he*t^ mutants than in *yw* controls (*t*-test: *P* = 0.0085). All experiments performed on 10- to 14-day-old flies.

As fatty acid transporters are thought to participate in fatty acid metabolism, we measured the effect of reduction in *dFatp* expression on triglyceride storage. Whole *yw;dFatp*^*het*^ males contain nearly fourfold higher triglyceride levels than age-matched *yw* males. Female *yw;dFatp*^*het*^ flies have nearly twofold higher triglyceride levels than controls. Precise excision of the *dFatp* insertion returns triglyceride levels to wild-type (Fig. 1a). *w[cs];dFatp*^*het*^ male flies display increased triglyceride levels (Fig. S2A,B), similar in magnitude to *yw;dFatp*^*het*^ flies. TAG was normalized to total protein to control for differences in cell number or volume. Both *yw;dFatp*^*het*^ and *w[cs];dFatp*^*het*^ males have elevated levels of protein (Fig. S2C,D). Triglycerides normalized to total protein levels confirm increased TAG in *dFatp*^*k10307*^ heterozygotes regardless of genetic background (Fig. S2E,F). Similarly, triglyceride levels were increased when an inducible RNAi construct targeting the *dFatp* locus was expressed in adipose tissue, albeit to a lesser extent (Fig. 1b).

The increased triglyceride storage could conceivably be explained by an increase in feeding behavior. To test this, we observed the feeding rate of *yw;dFatp*^*het*^ and *yw* flies using the proboscis extension method (Wong *et al*., 2009). *dFatp*^*het*^ flies instead display an approximately threefold reduction in feeding rate (Fig. 1c). Inducible RNAi against *dFatp* also produced a similar phenotype when expressed in adipose tissue (Fig. 1d).

Circulating glucose levels are marginally increased in both starved (Fig. S2G: *t*-test: *P* = 0.0476) and fed (Fig. S2H; *t*-test: *P* = 0.0073) female mutants. Glycogen levels in total fly homogenate are also mildly increased in both sexes (Fig. 1e). Additionally, *dFatp*^*het*^ males have significantly increased free fatty acid levels in circulating hemolymph (Fig. 1f).

### Lifespan extension

Changes in lipid metabolism are likely to have pleiotropic affects on animal physiology. We assessed the net effect of these changes on health and lifespan by measuring survival under standard laboratory conditions as well as several stress conditions.

During several survival experiments, we noted that background controls displayed substantial early-life mortality in several cases. The reason for this mortality is unclear. Recognizing that early control deaths might cause an overestimation of lifespan extension in mutants, we plotted and analyzed all survival curves with the first 20 days censored. In this way, we focus the statistical analysis and graphical display on the deaths that are related to aging rather than developmental or early-life events.

**Fig. 2:**
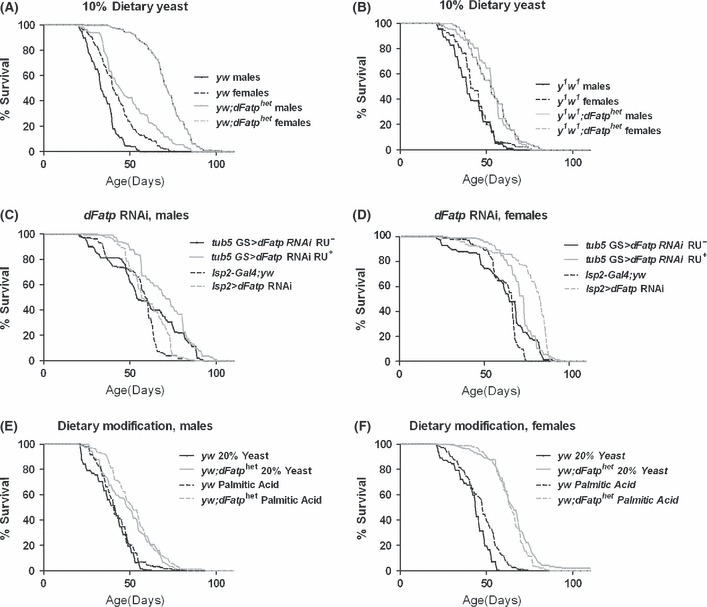
Reduction in *dFatp* expression extends lifespan and protects against high-nutrient diets. (A) Both male and female *yw;dFatp*^*het*^ flies live longer than *yw* flies on a 10% sucrose, 10% yeast laboratory diet (log-rank: males: *P* = 0.0183, females: *P* < 0.0001). (B) Both male and female *y*^*1*^*w*^*1*^*;dFatp*^*het*^ flies live longer than *y*^*1*^*w*^*1*^ flies on a 10% sucrose, 10% yeast laboratory diet (log-rank: *P* < 0.0001 for both). (C, D) *dFatp* RNAi flies of both sexes live longer than background controls on a 10% sucrose 10% yeast diet. *dFatp* expression was knocked down in adult flies ubiquitously (*tub5 GS-gal4*) and in adipose tissue (*lsp2-gal4*) (log-rank: *tub5 GS* *>* *dFatp RNAi* males: *P* = 0.0017, *lsp2Gal4* males: *P* = 0.0002, *P* < 0.0001 for all females). (E, F) Both male and female *yw;dFatp*^*het*^ flies aged on high-yeast or high-fatty-acid diets live longer than *yw* controls (log-rank: *P* < 0.0001 for all). Both yeast and palmitic acid were supplemented to 20% weight/volume. *n* ≥ 140 for all survival analyses. All statistical analysis here and in subsequent figures was performed with the first 20 days of life censored to remove deaths related to early mortality or developmental effects. Complete data can be visualized in the mortality curves shown in supplemental data and in accompanying life tables.

Survival was measured in three different backgrounds on 10% yeast, 10% sucrose diet. Male survival was increased in *yw;dFatp*^*het*^ and *y*^*1*^*w*^*1*^*;dFatp*^*het*^ backgrounds (Fig. 2a,b), but male lifespan was not significantly increased in a second *yw;dFatp*^*het*^ repetition (Fig. S3A) or in the *w[cs];dFatp*^*het*^ hybrid background (Fig. S3D). Survival of females was increased compared to background in *yw;dFatp*^*het*^ (two repetitions) and in *y*^*1*^*w*^*1*^*;dFatp*^*het*^ (Figs 2a,b and S3B,C log-rank for all female lifespans *P* < 0.0001). Fertility was not affected (Fig. S3E; *t*-test: *P* = 0.309). Comparison of female *yw;dFatp*^*het*^ survival to revertant lines was inconclusive, however, as female revertants lived longer than *yw* controls (Fig. S3B), indicating that the survival phenotype of the insertion is not fully rescued by precise excision.

*w[cs];dFatp*^*het*^ females lived dramatically longer than *w[cs]* females (Fig. S3C Iog rank: *P* < 0.0001). However, *w[cs];dFatp*^*het*^ females did not have a significantly extended lifespan when compared to the hybrid *w[cs];dFatp* revertant controls, indicating that lifespan extension in this hybrid background can be accounted for by heterosis.

Age-specific mortality plots are in agreement with left-censored survival results, with low aging-related mortality in all backgrounds where lifespan extension is significant (Fig. S4A and S6A–C).

We examined the effects of adult-specific reduction in *dFatp* by inducing ubiquitous expression of RNAi against *dFatp* after adult eclosion. Both male and female flies expressing RNAi against *dFatp* had an extended lifespan as compared to background controls (Fig. 2c,d log-rank for males *P* = 0.0017, females: *P* < 0.0001), indicating that adult-specific interference with *dFatp* expression is sufficient to generate lifespan extension. Because *dFatp* expression has been reported to be enriched in the fat body, we induced RNAi against *dFatp* in adipose tissue. Male and female flies expressing RNAi against *dFatp* in the fat body displayed a significant lifespan extension compared to background controls (Fig. 2c,d, log-rank for males *P* = 0.002, females *P* < 0.0001). A plot of age-specific mortality reveals that females expressing *dFatp* RNAi have low mortality across ages (Fig. S4D). Males, by contrast, are extended primarily due to high mortality in controls at early ages (Fig. S4C). Taken together, this indicates that *dFatp* RNAi treatment is effective at slowing aging in females.

Because Fatp proteins are thought to be responsive to circulating fatty acid content, we wondered whether increasing dietary fatty acid content would reverse the lifespan advantage conferred by the mutation. We tested this by supplementing our standard laboratory diet of 10% dietary yeast, 10% dietary sucrose with an additional 10% weight/volume of yeast. On this high-nutrient diet, both mutants and control flies lived less long than on our standard diet. However, *yw;dFatp*^*het*^ flies retained a substantial lifespan extension compared to the *yw* background (Fig. 2e,f; log-rank for males and females: *P* < 0.0001). Because yeast is a source of many nutrients, and not only lipids, we specifically supplemented the standard laboratory diet with 5 g L^−1^ per liter of palmitic or linoleic acid and measured survival of flies aged on these diets. Palmitic and linoleic acid were chosen because they represent the vast majority of fatty acid content in the brewer’s yeast used in this study (Mee *et al*., 1979). The increase in fatty acid percentage did not significantly alter media pH. *yw;dFatp*^*het*^ flies retain a statistically significant survival extension on a diet supplemented with excess palmitic acid (Fig. 2e,f; log-rank for males and females: *P* < 0.0001), or linoleic acid (data not shown; log-rank for males and females: *P* < 0.0001). Mortality plots reveal a trend toward reduced mortality across ages in both male and female mutants subjected to either dietary treatment (Fig. S4E,F).

In summary, we find that reduction in *dFatp* expression by genomic mutation confers female lifespan extension in two of three genetic backgrounds tested. In addition, two different RNAi treatments against *dFatp* expression confer lifespan extension. Males, by contrast, display a more inconsistent and generally smaller lifespan extension when *dFatp* expression is reduced. Lifespan extension in neither sex is sensitive to increased dietary nutrient content, including increased fatty acid content.

### Stress resistance

**Fig. 3:**
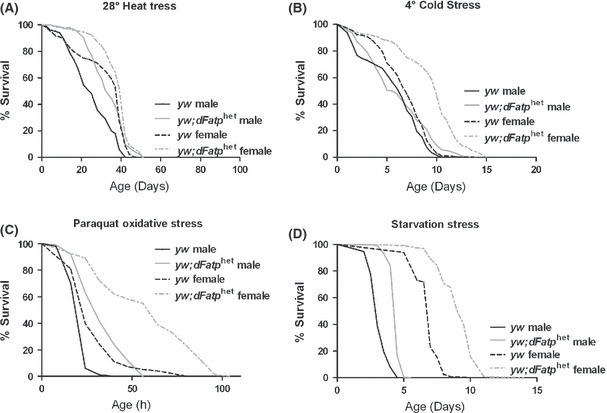
*dFatp*^*het*^ flies display an increased resistance to multiple stresses. (A) Male *yw;dFatp*^*het*^ flies live longer than *yw* flies under heat stress (28 °C) (log-rank: *P* < 0.0001). (B) Female *dFatp*^*het*^ flies live longer than *yw* flies under cold stress (4 °C) (log-rank: *P* < 0.001). (C) Both male and female *dFatp*^*het*^ flies were more resistant to paraquat than *yw* flies (log-rank: *P* < 0.0001). (D) Male and female *dFatp*^*het*^ flies live longer than *yw* under agar-only starvation conditions (log-rank: *P* < 0.0001).

Lifespan extension under standard laboratory conditions may or may not involve increased resistance to more extreme conditions. Therefore, we tested the survival of *yw;dFatp*^*het*^ flies under a variety of stressful conditions. Mutant males experience a statistically significant lifespan extension when aged at 28 °C, a condition of chronic heat stress (Fig. 3a; *P* < 0.0001, Fig. S4A). In the opposite case, when animals were aged at 4 °C, female, although not male, mutants exhibited a significant extension of lifespan (Fig. 3b; *P* < 0.0001 for females, Fig. S4A).

In order to test resistance to oxidative stress, we treated adults with paraquat and measured survival. Both male and female *yw;dFatp*^*het*^ flies were long-lived compared to controls under paraquat treatment (Fig. 3c; *P* < 0.0001, Fig. S4A). In order to test resistance to starvation conditions, we aged *yw;dFatp*^*het*^ flies and controls on agar-only food conditions. Both male and female *yw;dFatp*^*het*^ flies were long-lived compared to controls (Fig. 3d; *P* < 0.0001 S4D). A potential caveat of the paraquat study is that reduced feeding rate in mutants may contribute to resistance by reducing ingestion of paraquat.

We conclude that reduction in *dFatp* expression is sufficient to provide protection simultaneously against multiple stresses.

### Cardiac function

Mutations or interventions that extend lifespan and stress resistance may have either positive or negative effects on general vigor or function of major organ systems. Flies aged on a diet high in fat accumulate lipid in the heart and show symptoms of impaired cardiac function similar to human lipotoxic cardiomyopathy (Birse *et al*., 2010). As *dFatp*^*het*^ flies have abnormally high triglyceride storage (Figs 1a and S2A) and high free FA in their circulation (Fig. 1f), we tested various indices of cardiac performance to assess potential impairment.

**Fig. 4:**
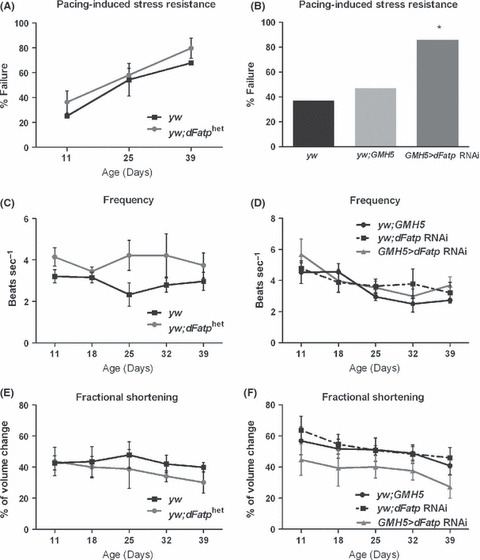
Reduction in *dFatp* in the heart impairs cardiac performance. (A) *yw;dFatp*^*het*^ flies have an increased sensitivity to cardiac pacing across 5 weeks of age compared to *yw* (two-way anova: *P* < 0.0001). (B) Knockdown of *dFatp* specifically in the heart (*GMH5* > *dFatp* RNAi) causes an increase in pacing-induced failure rate of 12-day-old flies (two-way anova: *P* < 0.0001). (C) Cardiac frequency is increased in *yw;dFatp*^*het*^ flies across ages when compared to *yw* controls (two-way anova: *P* < 0.0001). (D) Knockdown of *dFatp* specifically in the heart does not significantly alter cardiac frequency (two-way anova: *P* = 0.5649). (E) *yw;dFatp*^*het*^ hearts display a reduced fractional shortening across ages (two-way anova: *P* = 0.0343). (F) Knockdown of *dFatp* in the heart causes a reduction in fractional shortening across ages compared to background controls (two-way anova: *P* < 0.0001). *n* ≥ 10 flies for all cardiac analyses. Both male and female flies were analyzed and were not found to be significantly different. Therefore, male and female data were combined for all graphs in Figure 4.

Using an external heart pacing method (Wessells & Bodmer, 2004), we tested cardiac stress resistance. The rate of cardiac failure in response to pacing stress is significantly higher in *yw;dFatp*^*het*^ flies than in *yw* flies across ages (Fig. 4a; two-way anova: *P* < 0.0001). The slope of age-related decline, however, is identical between mutant and wild-type flies (multivariate regression; genotype-by-age effect: *P* = 0.7714), consistent with an acute defect rather than a progressive age-related impairment. To assess whether cardiac phenotypes of *dFatp* mutants are tissue-autonomous, we used a cardiac-specific driver (*GMH5-Gal4*) to perform heart-specific *dFatp* knockdown. Such flies also display an increased failure rate when subjected to external electrical pacing (Fig. 4b; two-way anova: *P* < 0.0001).

The frequency of the heart rate is increased across ages in *yw;dFatp*^*het*^ flies (Fig. 4c; two-way anova: *P* < 0.0001), and hearts with reduced levels of *dFatp* display a reduction in fractional shortening (Fig. 4e; two-way anova: *P* = 0.0343), an indirect measure of cardiac output. Flies expressing *dFatp* RNAi specifically in the heart do not exhibit defects in frequency resembling those of the mutants (Fig. 4d), but do show similar defects in fractional shortening (Fig. 4f; two-way anova: *P* < 0.0001). In summary, *dFatp*^*het*^ hearts beat at a higher rate than wild-type flies, but exhibit weak contractions that are unlikely to pump as much hemolymph as those of wild-type flies. Cardiac-specific knockdown is sufficient to recapitulate fractional shortening and electrical pacing phenotypes, while the frequency defect may be regulated tissue non-autonomously.

### Rescue of cardiac phenotype by exercise

**Fig. 5:**
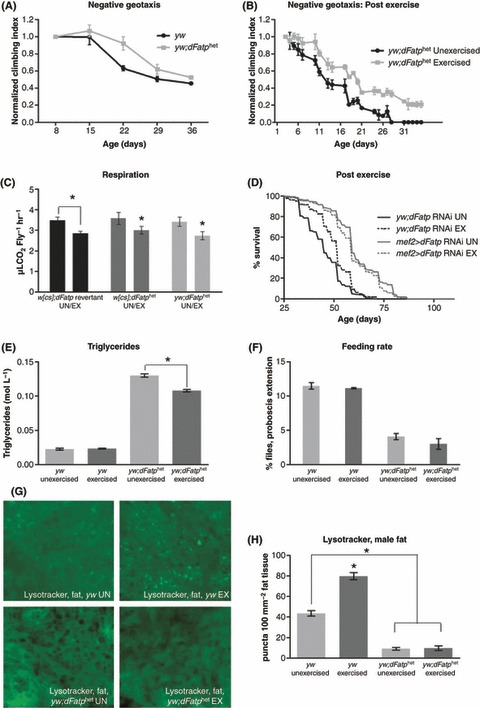
Endurance exercise rescues a subset of mutant phenotypes. (A) *yw;dFatp*^*het*^ flies display a significantly slower age-related decline in negative geotaxis ability compared to *yw* (Multivariate regression: genotype-by-age: *P* = 0.0019). Generally, male flies of all genotypes exhibit a stronger response to endurance exercise. Therefore, exercise experiments were conducted exclusively on male flies. (B) *yw;dFatp*^*het*^ flies show improved negative geotaxis ability during and following a 3-week time course of exercise training compared to unexercised siblings (Multivariate regression: treatment by age: *P* < 0.0001). (C) *w[cs];dFatp* revertant flies have significantly decreased CO_2_ production following exercise training (*t*-test: *P* = 0.013). *w[cs];dFatp*^*het*^ and *yw;dFatp*^*het*^ mutants have lower CO_2_ production than control flies following exercise training (two-way anova: *P* = 0.016, *n* ≥ 20). Respiration is not significantly different between genotypes (two-way anova: *P* = 0.961). (D) Flies with *dFatp* knockdown in the muscle/heart have extended lifespan compared to background control flies, whether exercised or not (log-rank: *P* < 0.0001). Survival was recorded following cessation of exercise program at 25 days old. (E) Triglyceride levels in mutant flies that were exercise-trained are significantly reduced when compared to unexercised siblings (*t*-test: *P* = 0.002). Triglyceride levels of control flies are not altered by exercise. (F) *yw;dFatp*^*het*^ exercised flies have a similar feeding rate in comparison with unexercised siblings. However, the mutants, whether exercised or not, have a reduced feeding rate when compared to either exercised or unexercised *yw* controls (*t*-test for each: *P* < 0.0001). (G) Representative micrographs of lysotracker staining in fat tissue of 3-week-old flies at 40 × magnification. (H) Quantification of lysotracker stain performed in male adipose tissue shows increased staining in wild-type *yw* flies following an endurance exercise program. Both exercised and unexercised *yw;dFatp*^*het*^ flies exhibit low adipose lysotracker staining compared to background.

*yw;dFatp*^*het*^ flies are not defective in negative geotaxis ability. Rather, they have a delayed age-related decline in this measure of mobility and vigor in comparison with controls (Fig. 5a; genotype-by-age: *P* = 0.0019). We have recently developed a *Drosophila* exercise training protocol that increases negative geotaxis and cardiac function in males (Piazza *et al*., 2009). We used this protocol to determine whether *yw;dFatp*^*het*^ males are capable of responding to endurance exercise. Indeed, mutant males demonstrate a significant degree of improvement in negative geotaxis following a 3-week training period of endurance exercise (Fig. 5b; treatment by age: *P* < 0.0001). This improvement is similar in magnitude to previous observations of multiple wild-type strains (Piazza *et al*., 2009).

Endurance exercise is known to alter respiration and oxidative efficiency in mammalian species (Coffey & Hawley, 2007), but the effect of chronic exercise on baseline respiration of *Drosophila* has not previously been examined. Therefore, we examined respiration of mutant and wild-type males, with and without an endurance exercise program. We find that *w[cs];dFatp* revertant flies decrease CO_2_ production following a 3-week exercise program (Fig. 5c; *t*-test: *P* = 0.0132). Both *yw;dFatp*^*het*^ and *w[cs];dFatp*^*het*^ flies have similar baseline CO_2_ production to control flies. Furthermore, *yw;dFatp*^*het*^ and *w[cs];dFatp*^*het*^ flies exhibit similarly decreased respiration following endurance exercise (Fig. 5c, two-way anova, *P* = 0.016), suggesting that *dFatp* mutation does not significantly alter either baseline respiration or the response of baseline respiration to chronic exercise in *Drosophila*.

Because flies with reduced *dFatp* expression respond readily to endurance exercise, we asked whether endurance training could rescue mutant phenotypes. First, we asked whether exercise altered the lifespan of *dFatp* mutants. To avoid complicating effects of injuries from exercise treatment itself, we measured lifespan beginning at 24 days, following the completion of 3 weeks of training. *dFatp* RNAi in muscle and heart resulted in lifespan extension, and this extension was unaffected by exercise treatment (Fig. 5d; log-rank: *P* < 0.0001), suggesting that exercise training in combination with *dFatp* reduction can improve both mobility and lifespan simultaneously. When mortality of the same flies was plotted, RNAi flies showed a distinct trend toward reduced mortality, particularly between days 20 and 80 (Fig. S6D).

The increase in triglyceride storage in *dFatp*^*het*^ flies was somewhat reduced by exercise training (Figs 5e and S3F), although not to wild-type levels. However, the reduced feeding rate of *yw;dFatp*^*het*^ flies persisted whether flies were exercised or not (Fig. 5f). It has been recently established that autophagy and lipid metabolism work in concert with modulate longevity pathways (Lapierre *et al*., 2011). In *Drosophila,* the effects of exercise training on autophagy and lipid metabolism have not been previously examined. Lysotracker-stained puncta in the fat of *yw* males increase following exercise training. However, *yw;dFatp*^*het*^ males exhibit very little lysotracker staining whether exercised or not (Fig. 5g,h).

**Fig. 6:**
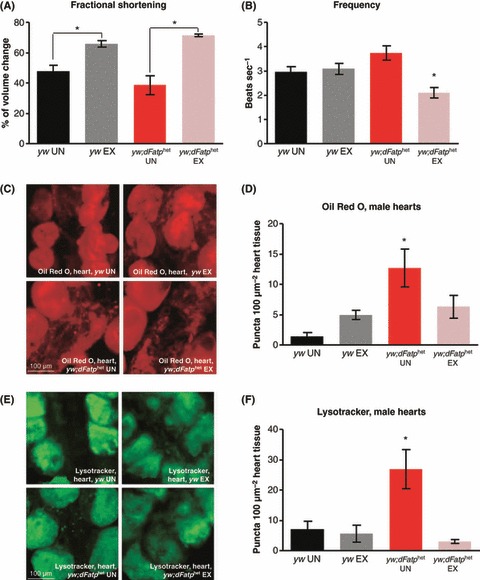
Endurance Exercise rescues cardiac phenotypes of flies with reduced *dFatp*. (A) Both exercise-trained control flies and exercise-trained *yw;dFatp*^*het*^ flies display increased fractional shortening compared to unexercised siblings at 3 weeks of age (*t*-test: *P* = 0.009, control flies, *P* = 0.002, *yw;dFatp*^*het*^). (B) Control *yw* flies exhibit no significant change in resting heart rate following exercise training (*P* = 0.714). Cardiac frequency is reduced in *yw;dFatp*^*het*^ flies following exercise training. (*P* = 0.049). (C) Representative 40 × micrograph of Oil Red O staining in *yw* and *yw;dFatp*^*het*^ hearts with and without exercise at 3 weeks of age. (D) Quantification of Oil Red O staining shows increased lipid in 3-week-old, unexercised *yw;dFatp*^*het*^ hearts (one-way anova: *P* = 0.004). Both male and female *yw;dFatp*^*het*^ flies were found to have increased baseline Oil Red O staining when compared to background controls. Males are depicted here. Following exercise, Oil Red O staining in *yw;dFatp*^*het*^ hearts returned to wild-type quantities (Dunnett pos *t*-test: *P* = 0.459 for exercised values when compared to unexercised control). (E) Representative 40 × micrograph of lysotracker staning in *yw* and *yw;dFatp*^*het*^ hearts, with and without exercise, at 3 weeks of age. (F) Both male and female *yw;dFatp*^*het*^ flies were found to have increased baseline lysotracker staining. Males are presented here. Quantification of lysotracker staining shows enhanced autophagy in 3-week-old, unexercised *yw;dFatp*^*het*^ hearts (one-way anova: *P* = 0.0006). Following exercise, lysotracker punctae in *yw;dFatp*^*het*^ hearts return to wild-type quantities (Dunnett pos *t*-test: *P* = 0.218 for exercised values when compared to unexercised control). *n* ≥ 10 flies for all stains.

Increased lipid content in the myocardium has been observed to impair fly cardiac performance (Birse *et al*., 2010). Endurance exercise has been shown to improve *Drosophila* heart performance in male flies (Piazza *et al*., 2009). Because exercise both reduces triglycerides and improves cardiac performance, we hypothesized that exercise could rescue cardiac impairment of *dFatp*^*het*^ flies. Further, we hypothesized that the mechanism of this rescue might be the reduction in lipid deposits in the heart muscle. Therefore, we placed *yw;dFatp*^*het*^ flies on an exercise training program and assessed them later for lipid levels in the myocardium and for rescue of cardiac performance. Cardiac pacing assays performed on mutant and wild-type populations prior to treatment produced results indistinguishable from those in Figure 4 (data not shown). However, exercised *dFatp*^*het*^ flies displayed a significant degree of rescue in fractional shortening (Fig. 6a; *t*-test: *P* = 0.002, Fig. S3G; *t*-test; *P* < 0.001), the critical defect displayed by the mutant flies. The increased frequency of heartbeats in *dFatp*^*het*^ flies is also reduced by exercise (Fig. 6b; *t*-test: *P* = 0.0049, Fig. S3H; *t*-test *P* = 0.028).

Because exercised males exhibit improvement in a subset of cardiac phenotypes, we applied Oil Red O staining to dissected hearts from *yw* and *yw;dFatp*^*het*^ males to determine whether reduction in lipids contributes to the mechanism of cardiac impairment and rescue. *yw;dFatp*^*het*^ flies display increased cardiac lipid staining, which is reversed following endurance training (Fig. 6c,d). *yw* male hearts have low Oil Red O staining with or without exercise (Fig. 6c,d, *P* = 0.0042). *yw* males display low levels of lysotracker staining regardless of exercise (Fig. 6e,f). However, *yw;dFatp*^*het*^ flies have increased autophagy in heart tissue, and this phenotype is reversed following exercise (Fig. 6e,f, *P* = 0.0006).

## Discussion

### Fatty acid metabolism and lifespan

The impact of fatty acid metabolism in any organism reflects a balance between dietary intake, fatty acid oxidation, and packaging into triglycerides for long-term storage. Here, we examine various effects of reduction in the expression of a proposed fatty acid transporter, *dFatp*. Critically, the reduction in expression provided by the *dFatp*^*het*^ mutation is not total. Complete loss of *dFatp* is lethal or semi-lethal in both homozygous null mutants and animals that constitutively express RNAi against *dFatp* throughout development. A partial reduction in *dFatp* expression, however, provides lifespan extension, high exercise capacity, and resistance to multiple stresses.

Flies with reduced *dFatp* expression have high fat storage, perhaps providing a signal for reduced feeding rate. Increased fat storage may contribute to the enhanced stress resistance conferred by *dFatp*, particularly the enhanced resistance to starvation. While increased TAG levels have been previously correlated with lifespan extension [e.g., *chico* (Clancy *et al*., 2001)], it is not clear whether this reflects a causative effect.

*dFatp*^*het*^ flies are not impaired in their response to endurance exercise and, indeed, display a modest improvement in untrained mobility. We propose that the increased fat storage in the mutant flies becomes beneficial under stress conditions, when it could be mobilized to provide protection against starvation and cold and to provide additional energy during endurance training.

However, other aspects of physiology do not benefit from these changes. In particular, cardiac performance is substantially impaired when *dFatp* expression is reduced, either globally or in the heart itself. Crucially, this impairment can be reversed by endurance exercise, without suppressing beneficial phenotypes. Various aspects of this model are discussed separately below.

### Feeding behavior

*dFatp*^*het*^ flies display a substantial reduction in feeding rate, accompanied by an increase in triglyceride storage. This observation is consistent with other recent reports in which mutations in worms or flies both reduce feeding rate and increase fat storage. For example, worms with a mutation in *daf-7*, which encodes a ligand of the TGF-β family, increase fat accumulation and reduce feeding rate (Greer *et al*., 2008). In flies, mutants for *AKHR*, which encodes a receptor for adipokinetic hormone, also display low feeding rate and high triglyceride storage (Bharucha *et al*., 2008). Taken together, these phenotypic observations suggest that fat itself may produce one or more signals to reduce feeding rate in adult invertebrates.

In addition to endocrine signaling from adipose tissue, neuronal signaling is likely to be required for this regulation to occur in adult flies. Potential candidates for such signals include serotonin, whose reduction is known to reduce feeding rate without lowering negative geotaxis ability in adult flies (Neckameyer *et al*., 2007), a combination of phenotypes reminiscent of those seen in *dFatp* mutants.

It is possible that reduced feeding rate may be causally mediating lifespan extension, perhaps by a dietary restriction (DR)-dependent mechanism. The phenotypic differences between DR and *dFatp* reduction, especially negative geotaxis, cardiac performance, and exercise response, argue against a DR-dependent mechanism. However, we cannot rule out the possibility that feeding rate reduction contributes to lifespan extension by inducing a subset of effects induced by DR.

### Cardiac functional impairment

Cardiac performance and lifespan are separable in *Drosophila* (Wessells & Bodmer, 2004; Piazza *et al*., 2009; Piazza & Wessells, 2011). Impairment of cardiac function is a well-characterized consequence of high levels of triglyceride storage in humans (Unger *et al*., 2010). The relationship of fat levels with cardiac performance in flies is less well understood. Flies fed a diet supplemented with coconut oil display impaired cardiac function (Birse *et al*., 2010), but mutations that increase triglyceride levels, such as *chico* (Clancy *et al*., 2001), can also provide substantial protection of cardiac function during aging (Wessells *et al*., 2004). In flies fed a high-fat diet (Birse *et al*., 2010) and in rodents overexpressing fatty acid transporter genes in the heart (Chabowski *et al*., 2007), accumulation of lipid deposits in the myocardium has been observed, leading to lipotoxic cardiac dysfunction. Here, we find that lipid levels in the heart are significantly increased in *dFatp*^*het*^ mutants. However, exercise rescues both cardiac performance and myocardial lipid content. These results are consistent with the hypothesis that cardiac functional impairment in these mutants is a result of lipotoxicity.

### Autophagy

In *dFatp* mutants, we observe abnormally high myocardial autophagy. In a rabbit model of myocardial infarction, compromised cardiac muscles exhibit increased autophagosome accumulation, which is ameliorated following exercise (Chen *et al*., 2010a). Following endurance training, *yw;dFatp*^*het*^ males exhibit a similar response. We have yet to determine whether autophagy has a causative role or is an indirect effect of high lipid storage.

In contrast to cardiac autophagy, *dFatp* mutants have low autophagy in adipose tissue whether exercised or not. Several interventions have been shown to modify autophagy levels in fat. These changes have variously correlated with either lifespan extension (Partridge *et al*., 2011) or lifespan reduction (Scott *et al*., 2004). In the case of the *dFatp* fat body, low autophagy and high TAG correlate with increased lifespan in mutants. Autophagy responds tissue-specifically to lipid levels to protect against lipotoxicity (Singh, 2010). In wild-type flies, exercise increases autophagy in the fat body but not the myocardium, and lifespan is unaffected. These findings indicate that in flies (i) autophagy in fat can vary highly without affecting lifespan; and (ii) exercise can modify autophagy tissue- and context-specifically.

### Sexual dimorphism of phenotypes

Sexual dimorphism has been frequently observed in single-gene mutations that extend lifespan (Bartke, 2011) and in environmental interventions such as DR (Chen *et al*., 2010b; Soh *et al*., 2007). Some mechanisms associated with sexual dimorphism are inherently female specific, such as the link between female infertility and enhanced lifespan. In the case of *dFatp*, fertility is normal, ruling this out as the mechanism of lifespan extension. In other cases, the mechanisms responsible for sexual dimorphism are not fully understood.

Here, we find that *dFatp*^*het*^ females have a significant lifespan extension in multiple backgrounds, whereas *dFatp*^*het*^ males have either a more modest lifespan extension or none at all, depending on background. Additionally, we observe substantially larger increases in TAG levels as well as differential increases in stress resistance in *dFatp*^*het*^ females. Conversely, male and female *dFatp*^*het*^ flies have similar cardiac abnormalities, reduced feeding rate, and altered autophagy levels. The correlation between sexually dimorphic phenotypes, such as TAG storage and stress resistance, and greater lifespan extension seen in females may suggest that TAG storage and stress resistance are important factors driving the lifespan extension.

Further questions remain regarding the mechanistic basis of lifespan extension associated with *dFatp* mutation. The relative contributions of fat storage, reduced feeding rate, and altered autophagy will require further experimentation to fully define the mechanisms of *dFatp* action. Another major question regards the cellular role of the dFatp protein itself. Localization of the protein under various conditions and the effect on phenotypic outputs in the fly model may have particular significance to understanding the cellular role of dFatp.

## Experimental procedures

### Real-time polymerase chain reaction

cDNA was prepared from 7 to 10 whole, day-old male flies. Total RNA was extracted using Trizol (Invitrogen, Carlsbad, CA, USA) for each genotype. At least three independent RNA extractions were prepared for each sample. Relative message abundance was determined by amplification and staining with SYBR Green I using an ABI 7000 SDS (Applied Biosystems, Carlsbad, CA, USA). Expression of Rp49 and corresponding control *y*^*1*^*w*^*67*^*c*^*23*^ flies were used for normalization. Differences between genotypes were assessed by *t*-test or nested anova.

Primer sequences are listed below.

dFatp
Forward: 5′-AGAAACACCGAGTGCGTCTG-3′
Reverse: 5′-CCACCGTGTTGTCATGATTC-3′
CG7384
Forward: 5′-AAACCGACAGTTCTGGAAG-3′
Reverse: 5′**-**GCAGGAACTGACGATTGATG-3′
Lrr47
Forward: 5′**-**CCGCTGGTTAAATTCGAGTC-3′
Reverse 5′**-**ATCCGTAGATCCTCGACAGC-3′
Myo31DF
Forward: 5′**-**CTGGGTGGGTGATTATCTGG-3′
Reverse: 5′-AGCCTGCTTGTTGAAATGGT-3′
Rp49
Forward: 5′-ACTCAATGGATACTGCCCAAGA-3′
Reverse: 5′-CAAGGTGTCCCACTAATGCATA-3′
*dFatp* insertion
Forward: 5′-TGAACCTCGCACTCGGCTGAT-3′
Reverse: 5′-TTGGACAACTATGCGAACAGC-3′

### Triglycerides

Following collection, treatment, and aging, five female or eight male flies were weighed and homogenized in 500 μL of 0.05% PBS/Triton X buffer. Following lipase inactivation and centrifugation, the supernatant was added to 10 volumes of preheated (37 °C) Thermo Infinity Reagent (#TR22321; Thermo Electron Corp, Waltham, MA, USA). Absorbance of 520 nm was determined following 10 min of incubation with agitation at 37 °C. Resulting triglyceride measures were normalized per fly or per μg total protein. Each data point is based on at least three replicate measures.

### Feeding rate

After collection and mating as described in fly stocks and maintenance, 10-day-old adult flies were housed in vials of standard food (10% sucrose/10% yeast). Flies were separated into vials of five and left to equilibrate for 1 h. The percentage of flies engaged in proboscis extension in each vial was scored as in Wong *et al*. (2009). Each vial was observed 10 times in a double-blind fashion. To assess the amount of food consumed, 10-day-old adult flies were fed standard food supplemented with 0.5% (w/v) FD&C Blue #1 (Spectrum Chemical Mfg. Corp., Gardena, CA, USA). Following exposure to dyed food, five female or eight male flies were homogenized in 200 μL PBS and cleared by centrifugation at 15 700 ***g***. After centrifugation, the absorbance of the supernantant was measured at 625 nm. Having established blue-dye feeding assays as a control, data collected from proboscis extension methods were analyzed by Student’s *t*-test.

### Glycogen

After collection and mating as described, 30 male or female flies were weighed and homogenized in 200 μL PBS/0.05 × Triton X. Homogenate was cleared by spinning for 2 min at 13 000 rpm. Twenty microlitre of fly homogenate was added to 1 μL amyloglucosidase at 0.1 U μL^−1^. Sample was incubated with agitation at 37 °C for 1 h. Absorbance of 340 nm was determined within 30 min following incubation. Resulting glycogen was normalized per milligram dry weight. Data were analyzed using Student’s *t*-test.

### Free FA

Free FA were assessed from the hemolymph of 25–30 male flies per genotype. Hemolymph was obtained by puncturing cuticle with a sterile needle and then ‘pulse’ centrifuging flies in an Eppendorf with a mesh screen to separate solid material. One microliter of hemolymph sample was combined with 49 μL BioVision Free Fatty Acid reagent (BioVision, Mountain View, CA, USA). Reagents were mixed according to manufacturer’s instructions following standardization preparation and acyl-coA synthesis. Reaction was allowed to incubate, protected from light, for 30 min at 37 °C. Absorbance of 570 nm was determined following reaction completion. Data were analyzed using Student’s *t*-test.

### Survival assays

Background genotypes utilized in this study frequently displayed unusually high early-life mortality. Because this early mortality is unrelated to aging, we have plotted all survival assays except for stress assays with deaths from the first 20 days censored. Differences in all survival curves were assessed following censoring using log-rank analysis, applied only to post-20-day data. To provide a clear illustration of age-dependent mortality differences, we also plotted age-specific mortality for all curves in their entirety (Supporting Information.

Prior to all experiments, fly cultures were maintained at a constant density for at least two generations. Fifteen virgin females and five males were mated in 300-mL bottles with 50 mL standard 10% sucrose 10% yeast unless otherwise described. Adult progeny were synchronized by collecting within 2 h of eclosion, over a 24-h time period. Groups of 20 age- and sex-matched flies were immediately transferred into narrow polypropylene vials containing 5 mL of appropriate food medium. Food vials were changed every second day at which time dead flies were removed and counted. Flies were housed in a 25 °C incubator on a 12:12 light/dark cycle at 50% relative humidity.

For starvation resistance, flies were collected and housed as described and placed on agar-only food. Survival measurements were recorded twice per day in 12-h intervals. To assess survival on high-nutrient diet, standard 10% sucrose/yeast food was supplemented with an additional 10% weight/volume brewer’s yeast, dry palmitic acid, or dry linoleic acid. Survival measurements were recorded every second day.

Oxidative stress was induced by introducing 40 mm methyl viologen dichloride hydrate (Sigma-Aldrich, St. Louis, MO, USA) dissolved in 5% sucrose. Deaths were recorded three times per day in 8-h intervals. Temperature stress was assessed at either 4 or 28 °C. Cold survival was assessed twice per day in 12-h intervals. Before recording deaths from flies under cold stress, flies were given 1 h to recover movement at room temperature. Heat stress was recorded every second day.

### Respiration

CO_2_ production was measured using a flow-through respirometry system. Twenty-eight-day-old exercised and unexercised flies were immobilized 24 h before measurement by CO_2_ gas and separated into groups of five flies per sample. Samples were assayed in random order to evenly distribute variance between measurements. For each measurement, seven samples were transferred into 2-mL glass measurement chambers in a room kept under constant light at 25 °C, and one chamber was left empty as a blank reference. Chambers were consecutively flushed for 150 s at a flow rate of 90 mL min^−1^ with CO_2_-free, water-saturated room air through an 8-channel MUX flow multiplexer (Sable Systems International, Las Vegas, NV, USA). Flushing of all samples was consecutively repeated four times per measurement, resulting in 20 min intervals during which the chambers were sealed before the second, third, and fourth flushing. Integrated CO_2_ concentration over time was measured for all samples during the fourth flushing using a Li-7000 CO_2_/H_2_O Analyzer (Sable Systems International) and used to calculate CO_2_ output over time per fly. All *n* values were between 19 and 46. Results were analyzed using a two-tailed *t*-test (Prism; GraphPad Software, San Diego, CA, USA).

### Lysotracker

Adult flies separated by age, genotype, and/or treatment were dissected, ventral side up, in room temperature PBS. Having exposed the heart and fat bodies, partially dissected flies were rinsed 3 × in fresh PBS. Lysotracker green (Molecular Probes, Eugene, OR, USA) was diluted to 0.01 μm in PBS and applied to dissected preps for 1 min. Samples were washed three additional times in PBS. Stained hearts and fat bodies were subsequently removed and mounted in one drop of antifade reagent (Molecular Probes). Slides were imaged on an Olympus BX41 compound fluorescence microscope (Olympus, Center Valley, PA, USA) using a 40 × objective. Images were analyzed using Image (National Institutes of Health, Bethesda, MD, USA). A minimum of five samples were analyzed for each tissue type. Data were subjected to Student’s *t*-test following quantification.

### Oil Red O

Adult flies were dissected similarly to lysotracker staining with the following changes. Dissections were performed in room temperature PBS/0.05% Triton X and fixed in 4% paraformaldehyde/PBS for 10 min. Following fixation, samples were washed 3 × in PBS. Oil Red O (Sigma-Aldrich) was diluted 1:100 in isopropanol and applied to partially dissected preps for 20 min at room temperature. Following staining, samples were washed 3 × in ddH_2_O. Hearts were removed and mounted as previously described. Imaging and analysis were identical to lysotracker protocols. A minimum of 10 fly hearts were analyzed for each genotype/treatment. Baseline stains (before exercise) were performed in both male and female flies and exhibited similar cardiac Oil Red O phenotypes compared to wild-type controls. All exercise experiments were performed on males.

### Statistical analysis

Data for triglycerides, total protein, feeding rate, glycogen, free FA, fluorescent stains, and single-time-point cardiac analyses were analyzed by Student’s *t*-test. Survival analyses were conducted using log-rank test. Negative geotaxis, cardiac, and metabolic analyses traced over time were analyzed by two-way anova. Bonferroni pos *t*-test s were used to compare significantly different values. For exercise treatment, multivariate regression was used to assess treatment by age. All statistics were generated using GraphPad Prism software version 5 (La Jolla, CA, USA).

## Supporting Information

**Fig. S1** *dFatp^K10307^* heterozygotes exhibit specific reduction of *dFatp* expression.

**Fig. S2** Triglycerides are increased in *dFatp* heterozygotes in multiple wild-type backgrounds.

**Fig. S3** Reduction of *dFatp* expression extends lifespan in two of three genetic backgrounds.

**Fig. S4** Age-specific mortality plots from lifespan experiments in Figure 2.

**Fig. S5** Age-specific mortality plots from lifespan experiments in Figure 3.

**Fig. S6** Age-specific mortality plots from lifespan experiments in Figures 5 and S3.
