# Supplementary figures and images for: dFatp regulates nutrient distribution and long-term physiology in Drosophila
Source: Aging Cell. 2012 Dec;11(6):921–32. doi: 10.1111/j.1474-9726.2012.00864.x (PMC3533766; doi:10.1111/j.1474-9726.2012.00864.x)

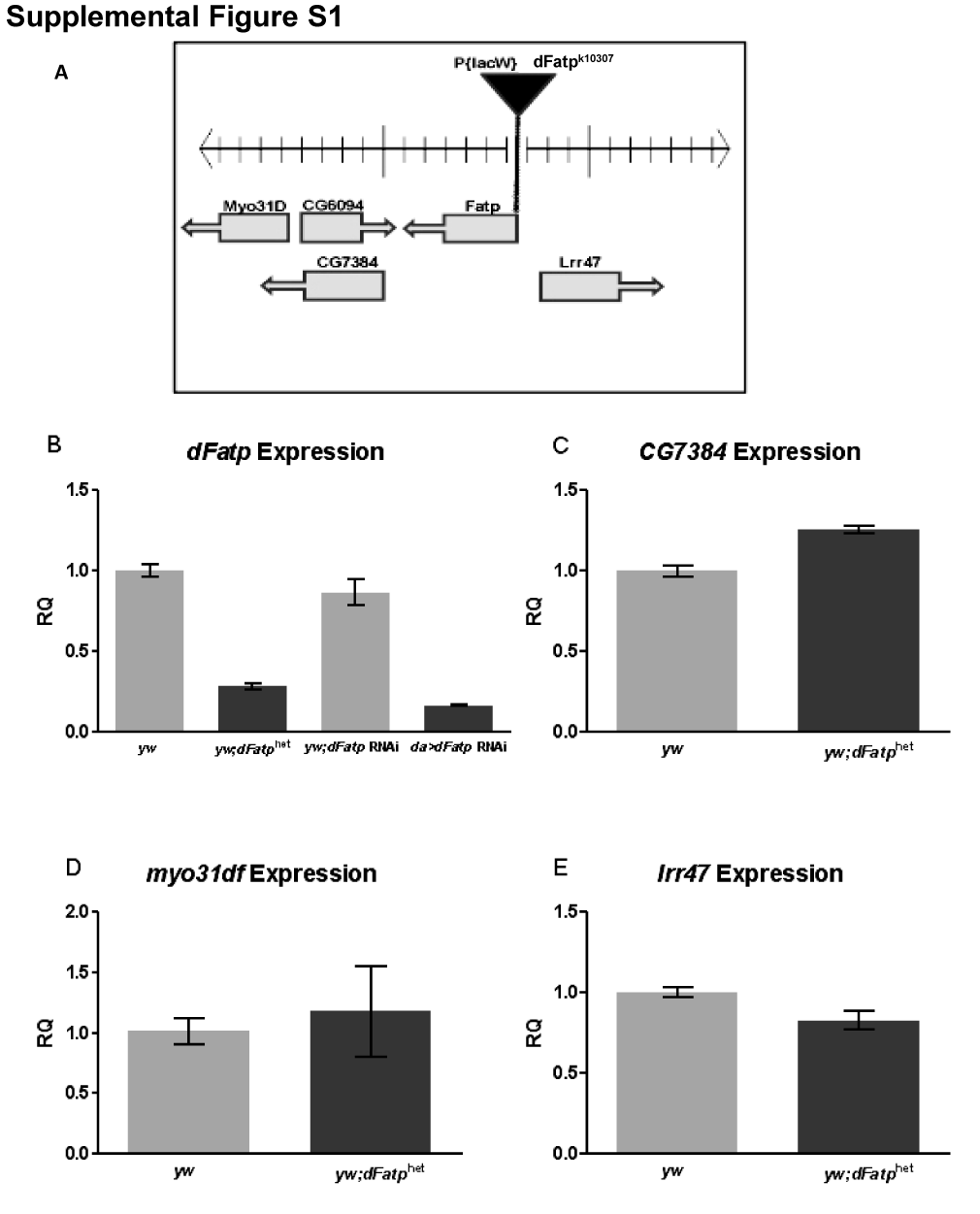

Supplement: Supplementary file 1 [file acel0011-0921-SD1.tif]

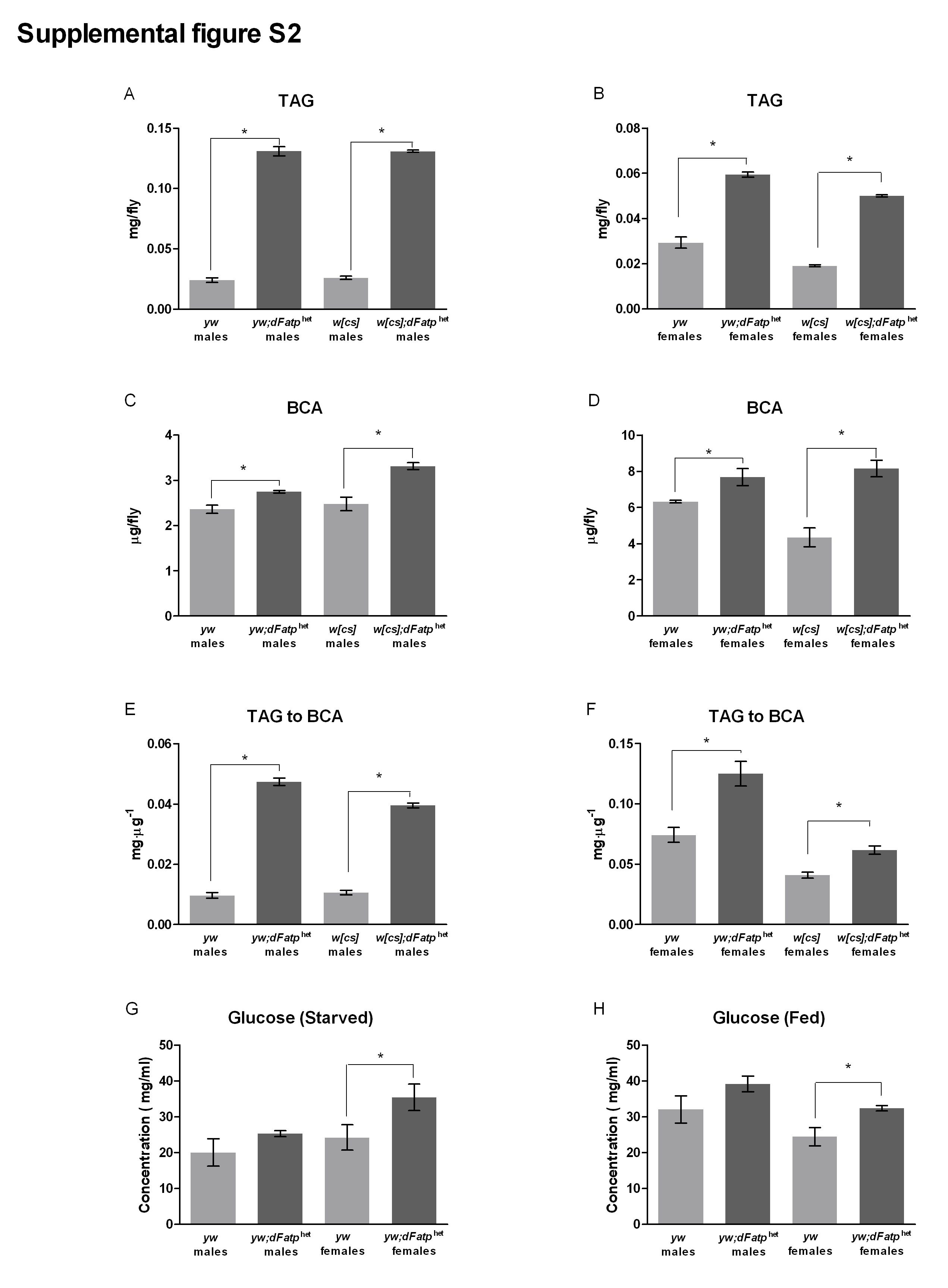

Supplement: Supplementary file 2 [file acel0011-0921-SD2.tif]

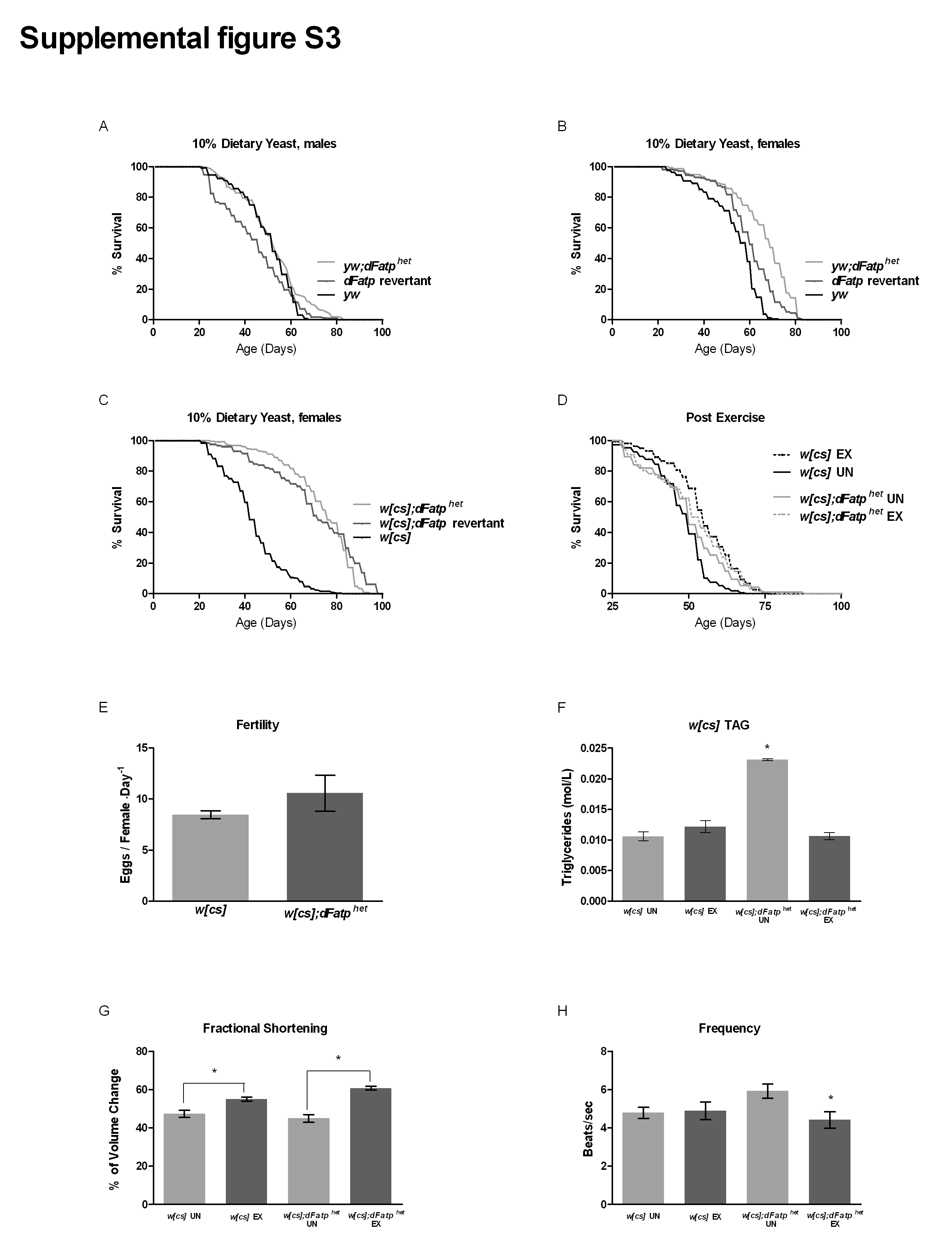

Supplement: Supplementary file 3 [file acel0011-0921-SD3.tif]

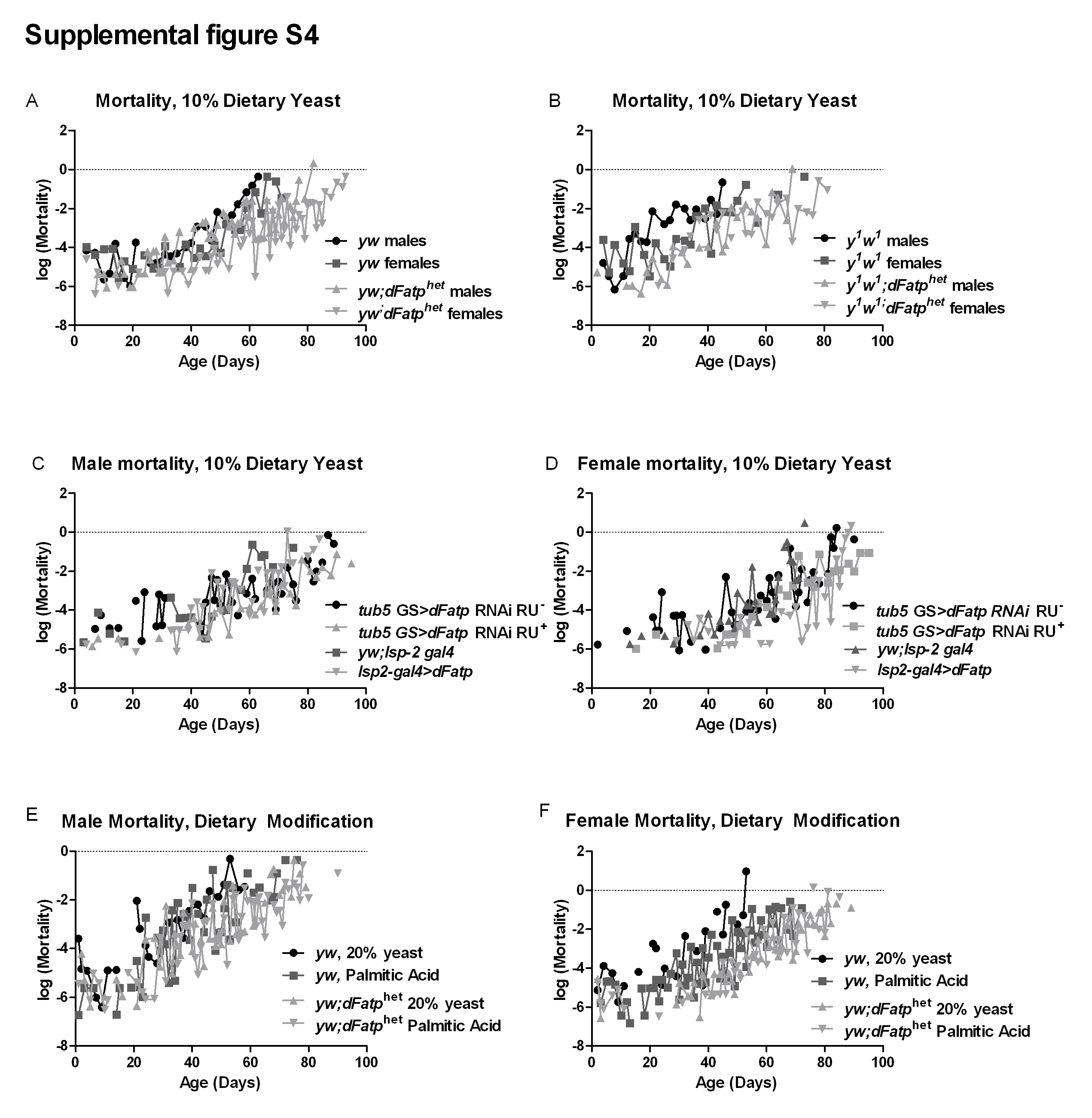

Supplement: Supplementary file 4 [file acel0011-0921-SD4.tif]

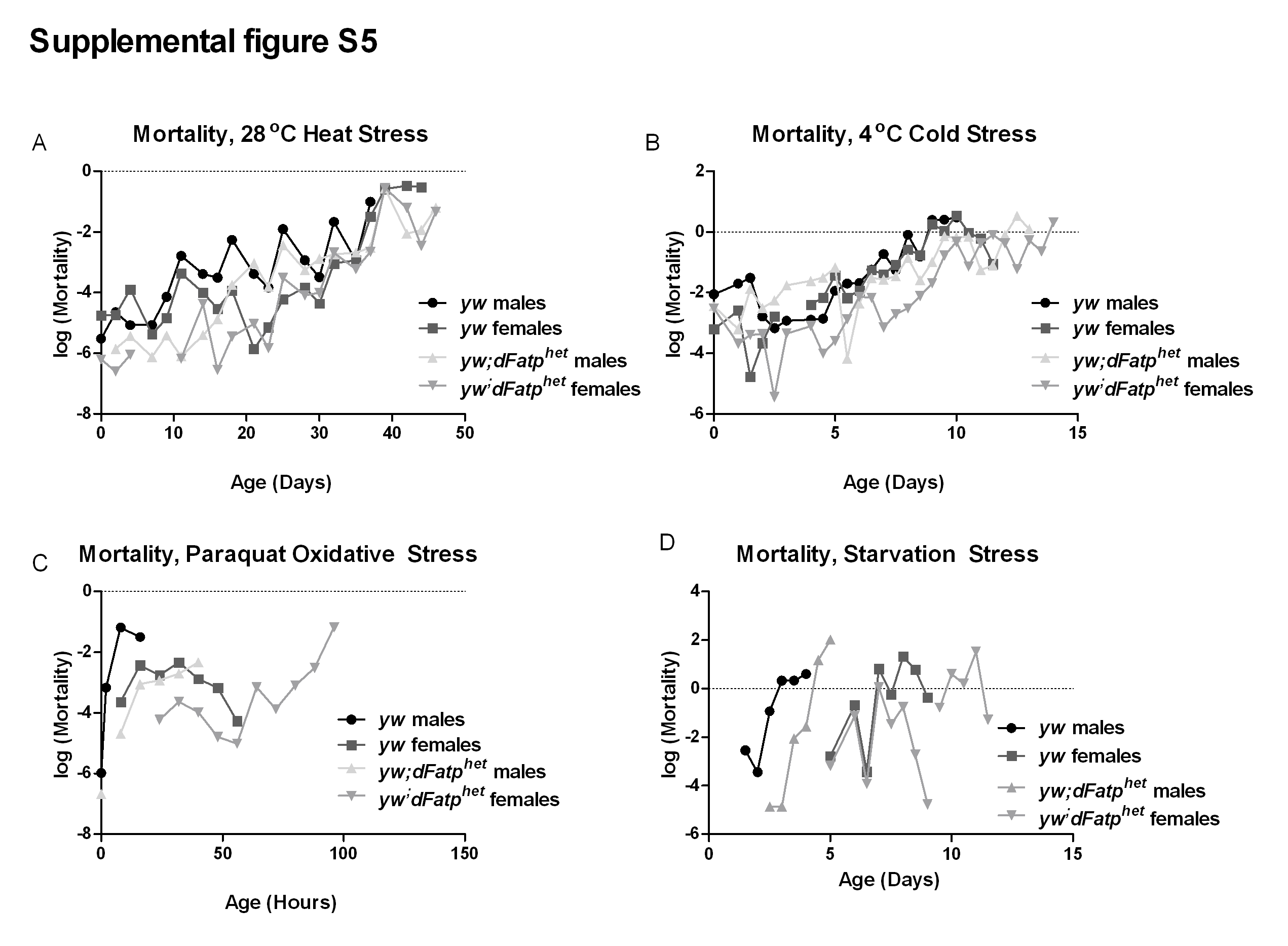

Supplement: Supplementary file 5 [file acel0011-0921-SD5.tif]

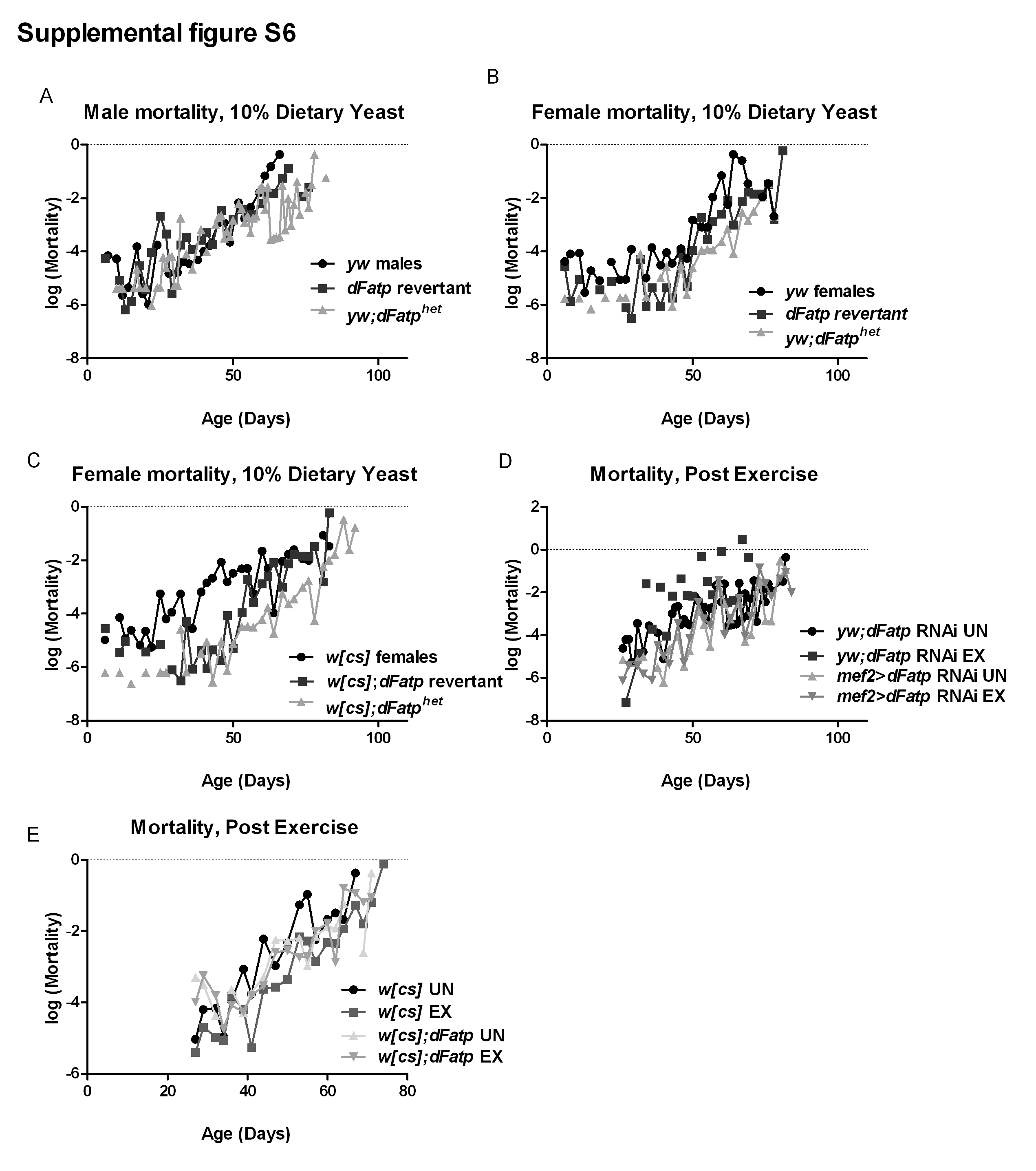

Supplement: Supplementary file 6 [file acel0011-0921-SD6.tif]
