# Supplementary material for: dFatp regulates nutrient distribution and long-term physiology in Drosophila
Source: Aging Cell. 2012 Dec;11(6):921–32. doi: 10.1111/j.1474-9726.2012.00864.x (PMC3533766; doi:10.1111/j.1474-9726.2012.00864.x)
Supplement: Supplementary file 7 [file acel0011-0921-SD7.doc]

**Supporting Information**

**Supplemental Figure 1. *dFatp^K10307^* heterozygotes exhibit specific reduction of *dFatp* expression.** (**A**) Gene map depicting P-element insertion site disrupting *dFatp****^K10307^***gene locus. Genes tested in C-E are also depicted. The insertion is not lethal over neighboring single gene insertions, including *Lrr47* and *Myo31D*, but is lethal over the deficiency Df(2L)J2, which disrupts the entire genomic region pictured. (**B**)*dFatp* expression is reduced by 72% in *dFatp^he^*^t^ flies when compared to *yw* wildtype control flies. Ubiquitous expression of RNAi against *dFatp* reduces *dFatp* expression by 83.4% in males. (**C-E**) Expression level of nearby genes in the genome is not significantly altered by the insertion in the *dFatp* locus (t-test: p>0.05 for all values).

**Supplemental Figure 2. Triglycerides are increased in *dFatp* heterozygotes in multiple wild-type backgrounds.** (**A, B**) TAG levels, measured in mg/fly, are increased in both *yw;dFatp^het^* and *w[cs];dFatp^het^* flies at 3 weeks of age (t-test: p<0.0001). (**C, D**) Total protein levels, measured in μg/fly, also increase in both *yw;dFatp^het^* and *w[cs];dFatp^het^* flies at 3 weeks of age (t-test: p=0.02, *yw;dFatp^het^*, p=0.007, *w[cs];dFatp^het^*). (**E, F**) TAG normalized to protein depicts a significant increase in triglyceride/protein ratio for both genotypes (p=0.008). (**G**) Haemolymph glucose concentration was significantly higher in starved *yw;dFatp^het^* mutant females (t-test: p=0.048), but not males (t-test: p=0.199). (**H**) Haemolymph glucose concentration was significantly higher in fed *yw;dFatp^het^* mutant females (t-test: p=0.007), but not males (t-test: p=0.124).

**Supplemental Figure 3. Reduction of *dFatp* expression extends lifespan in two of three genetic backgrounds.** (**A-B**) In a repetition of the experiment in Figure 2A, female, but not male *yw;dFatp^het^* flies live longer than *yw* background controls (log rank: p<0.0001 for females, p=0.07 for males). Comparison to revertant flies was inconclusive due to revertant flies living longer than *yw* controls. (**C**) Female *w[cs];dFatp^het^* flies have significantly increased lifespan when compared to *w[cs]* (log rank: p<0.0001). However, *w[cs];dFatp^het^* females are not significantly extended by log rank compared to progeny from a hybrid cross between revertants and *w[cs]*, indicating that lifespan extension in this background can be attributed to heterosis, rather than a direct effect of *dFatp* reduction. (**D**) Post-exercise lifespan of exercised and unexercised *w[cs];dFatp^het^* males is not significantly different from *w[cs]* controls, regardless of exercise traning. (**E**) Fertility of *w[cs]* females is not significantly different from that of *w[cs];dFatp^het^* flies (t-test: p=0.309). (**F**) Triglyceride levels in exercise-trained *w[cs];dFatp^het^* flies are significantly reduced when compared to unexercised siblings (t-test: p<0.001). Triglyceride levels of control flies are not altered by exercise. (**G**) Both exercise-trained control flies and *w[cs];dFatp^het^* flies display increased fractional shortening compared to unexercised siblings at 3 weeks of age (t-test: p=0.007, control flies, p<0.0001, *w[cs];dFatp^het^*). (**H**) Control *w[cs]* flies exhibit no significant change in heart rate following endurance training (p=0.842). Unexercised *w[cs];dFatp^het^* flies display increased heart frequency in comparison to wild-type flies (p=0.027). Frequency returns to wild-type rates following exercise training.

**Supplemental Figure 4. Age-specific mortality plots from lifespan experiments in Figure 2.** (**A**) Mortality data from experiment depicted in Figure 2A. *yw;dFatp^het^* males and females show reduced mortality after 50 days of age. (**B**) Mortality data from experiment depicted in Figure 2B. *y^1^w^1^;dFatp^het^* males and females show reduced mortality after 50 days of age. (**C**) Mortality data from experiment depicted in Figure 2C. Ubiquitous *dFatp* RNAi expression greatly reduces male mortality prior to 50 days of age. Adipose expression of *dFatp* RNAi modestly but consistently lowers male mortality across ages. (**D**) Mortality data from experiment depicted in Figure 2D. Either ubiquitous or adipose specific expression of *dFatp* RNAi dramatically reduces female mortality across ages. (**E-F**) Mortality data from experiments depicted in Figures 2E-F. *yw;dFatp^het^* males and females have reduced mortality after 50 days of age on diets supplemented with additional yeast or palmitic acid.

**Supplemental Figure 5. Age-specific mortality plots from lifespan experiments in Figure 3.** (**A**) Mortality data from experiment depicted in Figure 3A. *yw;dFatp^het^* males have reduced mortality throughout lifespan under 28°C stress. However *yw;dFatp^het^* females only have reduced mortality in the first 20 days. (**B**) Mortality data from experiment depicted in Figure 3B. *yw;dFatp^het^* females have dramatically reduced mortality under cold stress conditions. Males, however, have similar mortality to background controls under cold stress conditions. (**C**) Mortality data from experiment depicted in Figure 3C. *yw;dFatp^het^* males and females have substantially lower mortality than background controls when exposed to paraquat. (**D**) Mortality data from experiment depicted in Figure 3D. *yw;dFatp^het^* males and females have substantially lower mortality than background controls when exposed to starvation stress.

**Supplemental Figure 5. Age-specific mortality plots from lifespan experiments in Figure 5 and Supplemental Figure 3.** (**A**) Mortality data from experiment depicted in Figure S3A. *yw;dFatp^het^* males have similar mortality to background controls up until about 65 days of age, after which point they exhibit significantly reduced mortality. (**B**) Mortality data from experiment depicted in Figure S3B. *yw;dFatp^het^* females show reduced mortality after 50 days of age. (**C**) Mortality data from experiment depicted in Figure S3C. *w[cs];dFatp^het^* females have reduced mortality in comparison to *w[cs]* across ages, but not compared to revertants. (**D**)**.** Mortality data from experiment depicted in Figure 5D. Flies with reduced *dFatp* expression in muscle and heart have reduced mortality between 20 and 60 days of age whether exercised or not. (**E**) Mortality data from experiment depicted in Figure S3D. *w[cs];dFatp^het^* males have similar mortality to background controls between 20 and 70 days of age whether exercised or not.

**Supplementary Methods**

**Cardiac Assays**

Flies were aged as in survival assays. Once per week a minimum of 50 males and 50 females were removed from the cohort and subjected to electrical pacing as described in Wessells et al. (2004). The percentage of fly hearts that responded to pacing with either fibrillation or arrest was recorded as “% failure”. Percent failure is a marker for stress sensitivity (Wessells and Bodmer, 2004). Because sex did not significantly affect failure rate, combined data from 50 males and 50 females was analyzed by multivariate regression for age by genotype.

Video based assays were performed in whole flies as described in Piazza et al. (2009). Frequency was calculated by measuring the number of full beats divided by the length of time in seconds. Fractional shortening was computed by measuring the change in distance (mm) between the edges of the myocardial walls from maximum systole to maximum diastole divided by the heart width at maximum diastole. For each fly 10 measurements were taken and averaged. Graphs represent a minimum of 5 flies of each age, genotype, and/or treatment. xy plots were analyzed using multivariate regression for age by genotype/treatment. Single time point histograms from male flies at 3-weeks of age were analyzed using student t-test.

**Negative Geotaxis Behavior**

Adult flies were collected with light CO_2_ anesthesia within 2 hours of eclosion and housed in appropriate fresh food vials. Negative geotaxis was assessed in Rapid Negative Geotaxis (RING) assays in groups of 100-120 flies as described (Gargano et al., 2005). Flies were transferred to individual polypropylene vials in a RING apparatus and allowed to equilibrate for 1 minute. Negative geotaxis was elicited by sharply rapping the RING apparatus four times in rapid succession. The positions of the flies were captured in digital images taken 2s after eliciting the behavior. Digital images of the flies were analyzed using an algorithm developed by Scott Pletcher (climber). The relative distance climbed by each fly was converted into quadrants using Microsoft Excel. The performance of 20 flies was calculated as the average of four consecutive trials to generate a single datum. Flies were tested 5 times per week for 5 weeks to assess decline in negative geotaxis speed with age. Between assessments, flies were returned to food vials and housed until the following RING test.

**Endurance Exercise**

Exercise was performed as in Piazza et al. (2009). Cohorts of at least 200 male flies were collected under light CO2 anesthesia within 2 hours of eclosion and separated into vials of 20. Flies were then further separated into 2 large cohorts of 100 flies divided into exercised and unexercised groups. The unexercised groups were placed on the exercise training device, but were prevented from running by the placement of a foam stopper low in the vial. Exercised flies were placed in identical vials with normal cotton flugs. The exercise device drops the vials of flies every 15 seconds, inducing a repetitive, innate negative geotaxis response. Exercised flies are free to run to the top of the vial. A ramped program of gradually increasing daily exercise time was previously established to generate significant alterations in mobility that persist for at least 2 weeks following the 3 weeks of endurance training (Piazza et al., 2009).

**Total Protein**

Following collection, treatment, and aging, 5 female or 8 male flies were weighed and homogenized in 500μL of 0.05% PBS/Triton-X buffer. Supernatant was cleared by centrifugation at 13,000 rpm for 2 minutes. Protein assays were performed according to manufacturer’s instructions using 5μL fly homogenate combined with 200μL preheated (37°C) bicinchoninic acid reagent (Sigma-Aldrich, St. Louise, MO, USA). Absorbance of 562nm was determined following 30 minutes of incubation with constant agitation at 37°C. Resulting BCA measures were normalized per fly or per mg dry weight.

**Fly Stocks and Maintenance**

Except when noted in the main text, all fly lines were reared and aged at 25°C; 50% humidity with a 12 hour light-dark cycle and provided with a standard 10% yeast/10% sucrose diet. Brewer’s Yeast was obtained from MP Biomedicals (Solon, OH), 2.39% crude fat content. *y^1^w^67c23^;dFatp^k10307^*, *w^*^;wg^Sp−1^/CyO;∆2-3/TM6B*, *y^1^w^67c23^*, and *y^1^w^1^* were obtained from the Bloomington *Drosophila* Stock Center; *w[cs],* was obtained from Mike Grotewiel. *GMH5-gal4* flies are as described in Wessells et al. (2004). *mef2-gal4* was kindly provided by Cathy Collins. *lsp2-gal4* flies were kindly provided by Sean Oldham. *dFatp* RNAi line v9406 was obtained from the Vienna *Drosophila* RNAi Center.

All UAS, Gal4, and *dFatp^k10307^* insertions were backcrossed into the *y^1^w^67^c^23^* background for ten generations before analysis. The presence of the insertions was verified by the presence of a *w^+^* marker associated with the insertion. Precise excisions were generated by crossing *dFatp^k10307^* to *w^*^;wg^Sp−1^/CyO;∆2-3/TM6B* flies, selecting single males carrying transposase, crossing to *y^1^w^67^c^23^*, then scoring the F2 generation for eye color. Candidate revertant males were backcrossed into the *y^1^w^67^c^23^* background using standard techniques. Precise excision of the P-element was verified using genomic PCR. *yw;dFatp^het^* flies were used as a positive control.
